# Analysis of relative displacement between the HX wearable robotic exoskeleton and the user’s hand

**DOI:** 10.1186/1743-0003-11-147

**Published:** 2014-10-18

**Authors:** Marco Cempini, Alberto Marzegan, Marco Rabuffetti, Mario Cortese, Nicola Vitiello, Maurizio Ferrarin

**Affiliations:** The BioRobotics Institute, Scuola Superiore Sant’Anna, viale Rinaldo Piaggio 34, Pontedera, 56025 Italy; IRCCS, Fondazione Don Gnocchi, Biomedical Technology Department, via Capecelatro 66, Milan, 20148 Italy; IRCCS, Fondazione Don Gnocchi, Cardiac and Respiratory Rehabilitation Unit, via di Scandicci, Florence, 50143 Italy

**Keywords:** Rehabilitation, Orthosys, Wearability, Motion capture, Robotic, Exoskeleton

## Abstract

**Background:**

Advances in technology are allowing for the production of several viable wearable robotic devices to assist with activities of daily living and with rehabilitation. One of the most pressing limitations to user satisfaction is the lack of consistency in motion between the user and the robotic device. The displacement between the robot and the body segment may not correspond because of differences in skin and tissue compliance, mechanical backlash, and/or incorrect fit.

**Findings:**

This report presents the results of an analysis of relative displacement between the user’s hand and a wearable exoskeleton, the HX. HX has been designed to maximize comfort, wearability and user safety, exploiting chains with multiple degrees-of-freedom with a modular architecture. These appealing features may introduce several uncertainties in the kinematic performances, especially when considering the anthropometry, morphology and degree of mobility of the human hand. The small relative displacements between the hand and the exoskeleton were measured with a video-based motion capture system, while the user executed several different grips in different exoskeleton modes.

**Conclusions:**

The analysis furnished quantitative results about the device performance, differentiated among device modules and test conditions. In general, the global relative displacement for the distal part of the device was in the range 0.5–1.5 mm, while within 3 mm (worse but still acceptable) for displacements nearest to the hand dorsum. Conclusions over the HX design principles have been drawn, as well as guidelines for future developments.

**Electronic supplementary material:**

The online version of this article (doi:10.1186/1743-0003-11-147) contains supplementary material, which is available to authorized users.

## Introduction

In recent years there has been widespread use of robotic systems as advanced rehabilitation tools, both in clinical [1] and domestic environments [2]. In particular, attention has been focused on actuated orthoses (namely exoskeletons), thanks to their capability to convey powered assistance – either for patient treatment or user empowerment – at the single-joint level [3–6].

Exoskeletons require a shift in the robotic design paradigm towards ergonomics, comfort and safety: related innovative solutions includes series-elastic and variable-stiffness actuator technologies [7, 8], and novel symbiotic control strategies [9]. From the design point of view, endowing the robotic architecture with passive degrees of freedom (DoFs) is a widely used method to achieve enhanced wearability and compliance towards the user’s anthropometry [10, 11]. A recent example comes from the wearable hand exoskeleton HX [12, 13]: its design embeds enough DoFs to cover the variability of human hand gestures and grasps, and has been strongly refined to minimize weight and encumbrance. HX is a modular device, consisting of separable units for the index, thumb, metacarpus, wrist and forearm segments, and can be unlatched from its actuation system.

Having a multitude of DoFs, modules, and an actuation-detaching stage, HX is extremely usable and flexible [14, 15]. Its main drawback is that mechanical stability may be lessened: the main undesired effect is the presence of significant backlash in the human-robot physical interaction.

The aim of the present paper is therefore to analyze the stability of the physical interaction between the user and HX, critically addressing its design approach. The analysis has been conducted in terms of measured kinematic discrepancy between HX and the wearer’s hand; kinematics were acquired with a video-based motion capture system, during several types of grip and with different operative conditions of the robot. Optoelectronic systems have been widely used to measure hand and finger kinematics in both healthy participants [16–18] and patients [19], and also while they were wearing active exoskeletons [13, 20]. The feasibility of measuring such small displacements, as the ones in the present study, using a video-based motion capture system has been already demonstrated [21].

## Materials and methods

### HX hand exoskeleton

**Figure 1:**
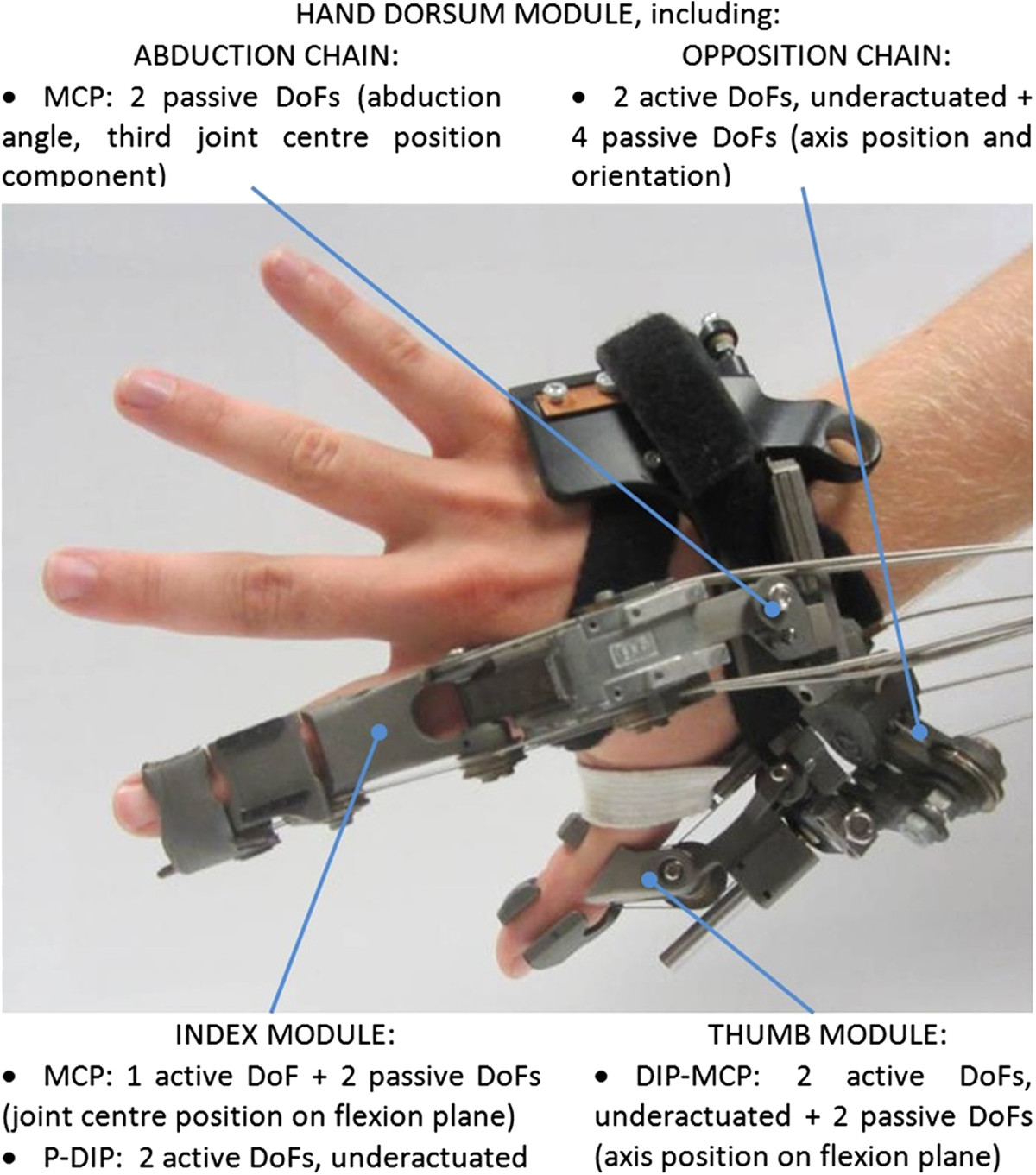
**Overview of the HX device.** The various HX modules and their kinematic chain architectures.

**Figure 2:**
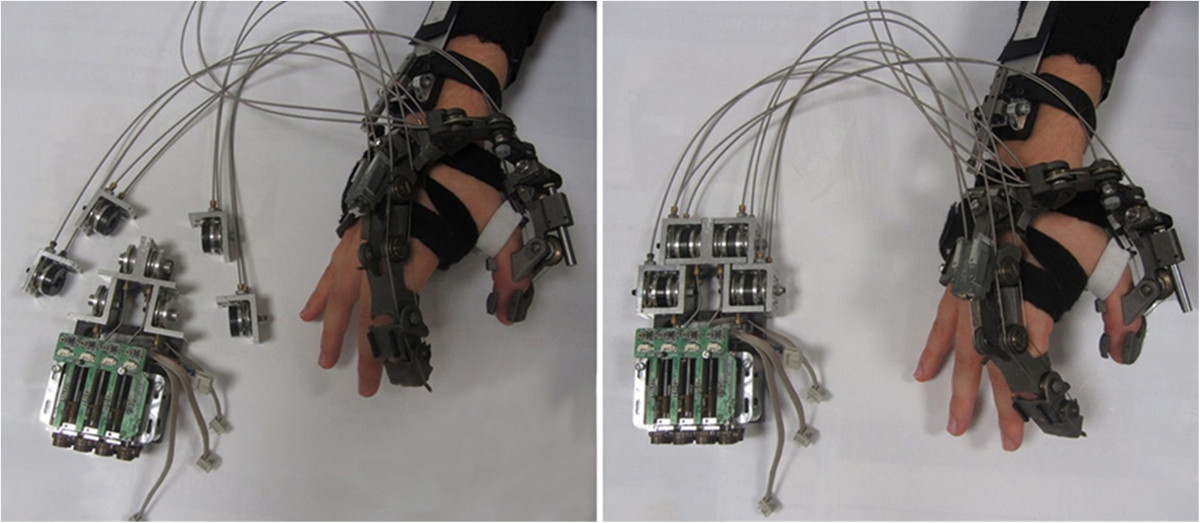
**Actuation.** Unlatched (left) and latched (right) actuation system, corresponding to the “passive” (when pulleys are detached, all DoFs are idle) and “active” modes. Forearm and wrist modules are also visible.

For the scope of the present study, we monitored three of HX’s wearable modules: index finger (IF), thumb (TH) and hand dorsum (HD) (Figure 1). HX transmitted a powered motion from external actuators to its modules without constraints between anatomical and robotic joint axes [11, 12]. In particular, IF and TH were attached to HD through articulated chains endowing passive DoFs for self-alignment and adaptation. Actuation was transmitted through tendon-sheaths pairs, which can be latched to the driving motor pulleys (Figure 2); latched or unlatched pulleys correspond to the “active” and “passive” HX operating modes.

### Data acquisition and experimental setup

**Figure 3:**
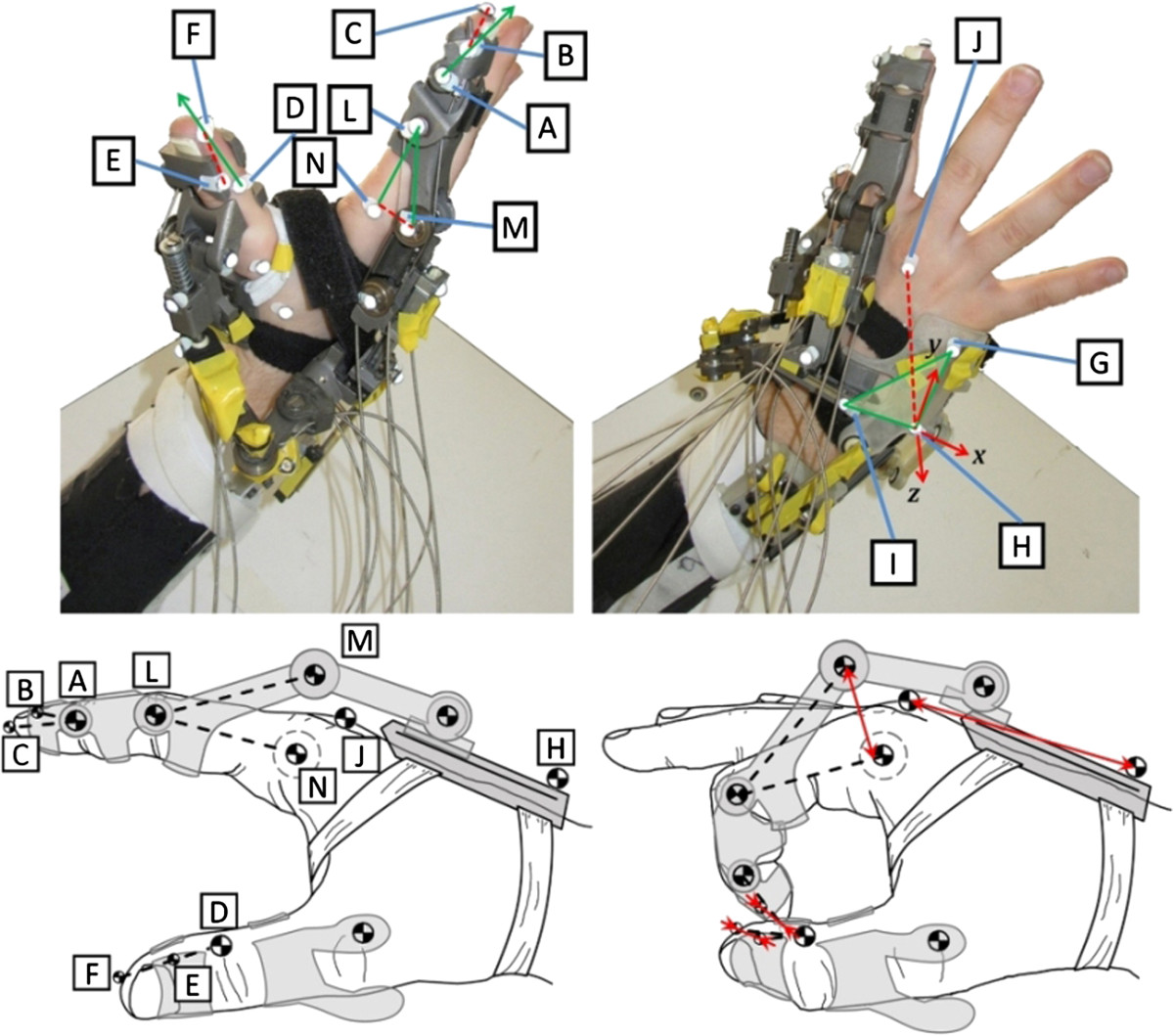
**Markers and relative displacement measures.** Positions of markers on the thumb and index finger (left) and on the hand dorsum (right). The figure represents the distances whose SD variability constitutes the analyzed numerical indexes.

An optoelectronic motion capture system (SmartD, BTS, Milan, Italy) was used to capture the 3D trajectories of hemispheric passive markers (5 mm diameter) moving in the calibrated volume (600×400×300 mm, *XYZ*). Seven TV cameras (640×480 resolution, 200 Hz sampling rate) were used, obtaining an average accuracy of 0.16 mm in the reconstruction of marker coordinates.Unlike other exoskeleton applications (e.g. lower limb), in this case it was nearly impossible to measure the user’s kinematics, since the hand segments were mostly covered by the HX itself. Hence, markers were positioned so as to address relative displacements between the device and the user at the distal (fingertip) and proximal (hand metacarpus) extremities. Respectively, we addressed the fingers phalanx-coaxial sliding within the TH and IF modules, and the phalanx-normal movement around the MCP joint (HD and IF modules). Specifically, the following positioning was used (Figure 3):

- TH: one marker on the IP joint medial side (*D*), one on the distal phalanx of TH (*E*) and one on the thumb tip (*F*);

b) IF-d (distal): two markers on the distal phalanx of IF (*A*, *B*), on the DIP joint and on the tip, respectively, and one on the user’s fingertip (*C*);
c) IF-p (proximal): two markers on the proximal phalanx of IF (*L*, *M*) and one on the user’s MCP axis (*N*);
d) HD: 3 markers on the dorsal shell of the HD (*G*, *H*, *I*) and one on the dorsal aspect of the hand (*J*), proximal to the knuckle of the middle finger.

Three different grips (palmar grasp, pinch and key grip), were tested in the following operating conditions:

1. *active:* the exoskeleton moved the hand of the participant, who was requested not to resist the robot’s movement;
2. *passive:* the participant moved their hand while wearing the unlatched exoskeleton.

Two additional tests served as references in the extreme conditions:

3. *passive, no-hand-movement:* global free motion of the upper arm, without moving fingers, hand or wrist;
4. *passive, hand-full-opening:* the participant fully opened their hand, reaching a complete extension of the fingers;

minimum and maximum relative movements are expected in these two conditions, respectively. Five trials were performed for each condition, and for each participant. Six healthy adult volunteers participated on the experiments, with their age, weight and height being 39.5 ± 9.1 years, 69.7 ± 7.5 kg, and 1.73 ± 0.05 m (mean ± SD) respectively. All the participants gave written informed consent approved by the Ethics committee of the IRCCS Don Carlo Gnocchi Foundation.

### Data elaboration

**Figure 4:**
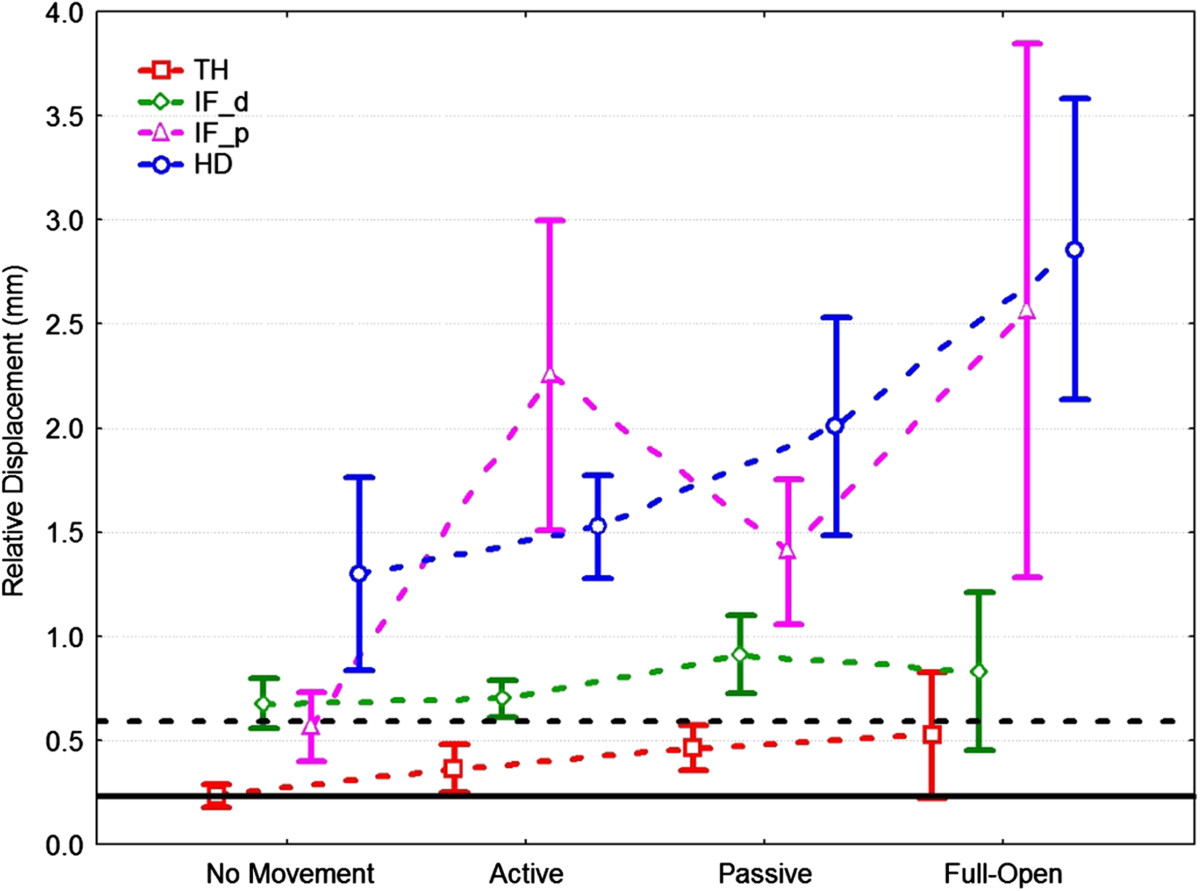
**Relative displacement indexes for different modules and condition.** Vertical bars represent the 0.95 confidence interval (corresponding to CI-95%) of the HX modules displacement. Horizontal black lines represent noise level (solid is mean value, dotted is the CI-95% upper limit value).

**Figure 5:**
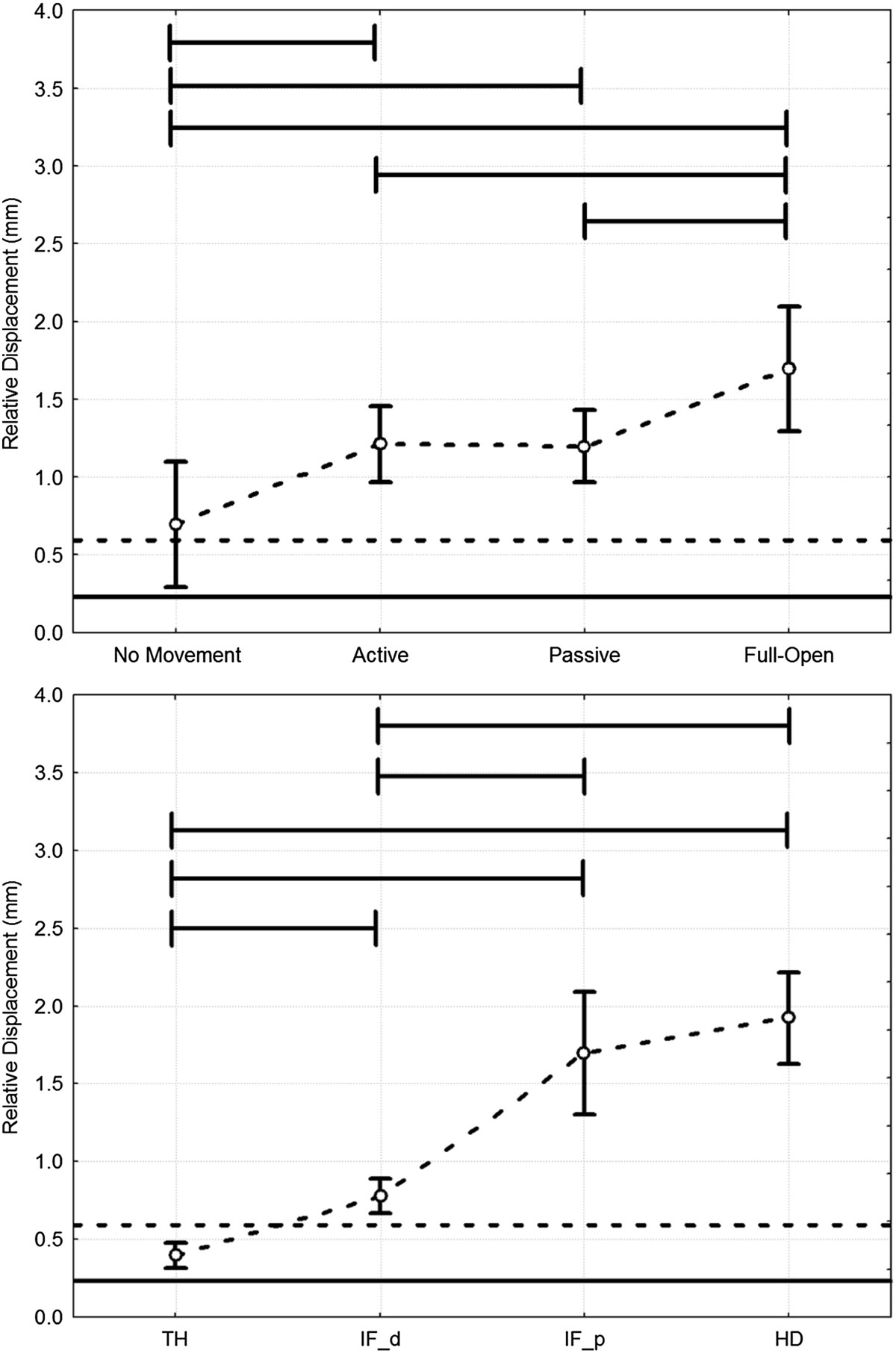
**Statistical analysis.** Results of statistical analysis (ANOVA, *p*<0.05; Fisher LSD post-hoc): effect of condition (top) and of HX module (bottom). Statistically significant differences among conditions and modules are marked with horizontal lines.

3D marker coordinates were reconstructed using the standard software associated with the motion capture system (Smart Tracker, BTS, Italy). Post-processing was performed with ad-hoc software developed in the MATLAB environment (MathWorks, Natick, MA) and included the following steps. The relative displacements of the HX modules were computed as (Figure 3):

- TH: standard deviation of the relative displacement between markers *E* and *F* as projected on the longitudinal axis of the distal phalanx (*D*−*F* versor);
- IF-d: standard deviation of the relative displacement between markers *B* and *C* as projected on the longitudinal axis of the distal phalanx (*A*−*B* versor);
- IF-p: standard deviation of the normal distance of marker *M* from the index proximal phalanx (segment *L*−*N*);
- HD: the quadratic mean of the standard deviations of the 3D coordinates of marker *J* in the dorsum local reference frame (origin in marker *H*);

Ideal values (a totally compliant exoskeleton) of these indexes were expected to be null. The relative displacement among markers placed on the same rigid frame, averaged over all conditions, was taken as the noise level of the experimental setup. An repeated measure ANOVA (*p*<0.05) was performed using Fisher’s LSD post-hoc comparisons to evaluate the differences between conditions and between TH, IF-d, IF-p and HD. Figure 4 shows the overall displacement indices for the different tests, and the noise average level (mean = 0.23 mm) and upper limit (CI-95% = 0.59 mm). Figure 5 shows the analysis, with ANOVA results indicated by horizontal lines joining statistically different data-sets.

## Results

Statistical analysis showed that both conditions and hand segments had a significant effect on HX wearing stability (Figure 5). The HD and IF-p showed the greatest displacement, while the IF-d and the TH were respectively just above and always within the noise band. As for the conditions, no-hand-movement and hand-full-opening showed the smallest and largest values respectively, with the two functional tests in between.

**Figure 6:**
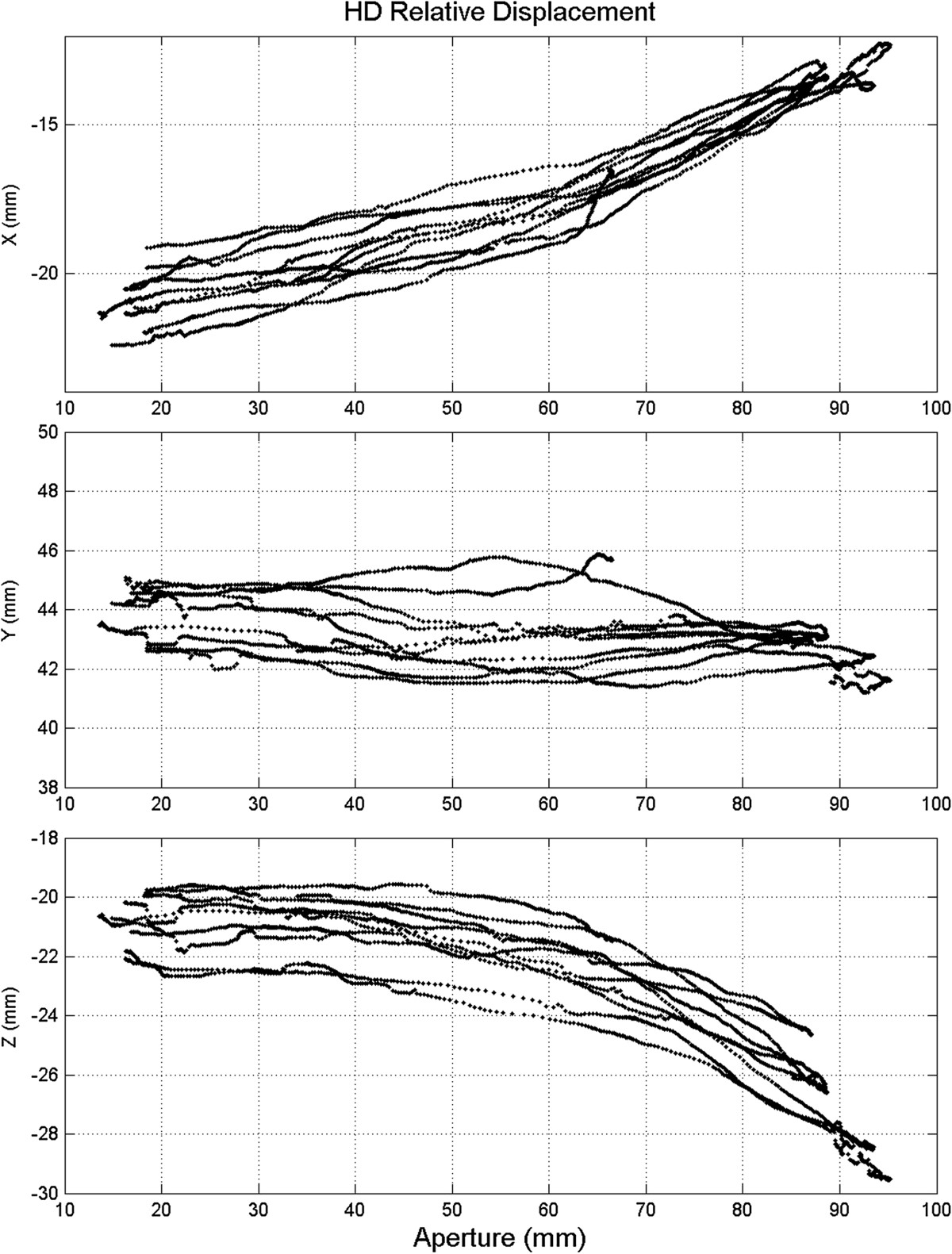
**HD-user relative displacement as function of hand aperture.** Example of *x*, *y* and *z* components (in mm) of the relative displacement between the HD module and hand dorsum, as a function of grip aperture amplitude during the hand-full-opening task.

IF-p was the only module showing larger “active” mode displacements than “passive” ones. HD relative displacements took into account the variability of the length of the vector joining the *J* and *H* markers; while the other measures were projections of marked vectors along given directions, the HD one included the three spatial components *x*, *y* and *z*, Figure 3. An additional analysis was performed on the HD results for the hand-full-opening test (the test with higher displacement). Figure 6 shows the relationships between the components of the relative movements and the grip aperture amplitude, the latter of which defined as the distance between the fingertip markers (*C* and *F*) for one of the six participants (representative of the others).

## Discussion and conclusions

Relative movements between an exoskeleton and the body segments are an indicator of user discomfort and lower robot effectiveness. The results of the present study showed that the stability of HX on the hand is optimal for the distal extremities of the actuated modules, even during the prospective therapeutic use of the device, where the robot drives the hand in executing grasps (“active” mode).Referring to the TH and IF-d plots in Figure 4, the difference between “active” and “passive” can be explained as a drawback of the tendon-driven actuation, which impedes completely transparent behavior because of friction losses in the cable routing. However, the minimal difference between the various modes for IF-d and TH means that HX grasp performance is good, i.e., the robot is stable in driving the fingertip towards closure.

On the contrary, IF-p showed higher displacements in the “active” mode than in the “passive” mode, though the difference was not significant (overlapping CI-95% intervals). This was also because of the sheaths-cable transmission system that, when actuated, pushes the structure while pulling the tendon, generating the revolving torque that is ultimately transmitted to the finger joints. The IF-p values are higher than the IF and TH ones; however, it is worth noting that the IF-p measure highly amplifies the effects of relative unfitting and local backlash in the first phalanx, because of the long geometric distance *LM*. Moreover, IF-p is the only measure *orthogonal* to the direction of the hand bones (Figure 3 – HD as well is related to a vector almost lying parallel to the metacarpus), hence it strongly emphasizes misalignment effects. We can state that IF-p represents the worst point for HX-user relative displacement, while IF-d and TH are effective evaluations of the physical grasping stability.

“Active” IF-p measurement remained confined in the 3 mm range (fairly acceptable; since this measure did not depend upon phalanx-parallel translation, but only on phalanx-normal distance variation, we can link it to an angular difference between the HX first phalanx and the user’s one, i.e. to a discrepancy between the MCP joint flexion angles. From the reference exoskeleton values of the *LM* distance and the $\hat{\text{MLN}}$ angle (40 mm and 35°), variations of 3 mm in the *MN* projection correspond to a maximum error of ± 5°.

Another critical segment is the HD module, likely because the hand metacarpus is anatomically complex, and it is difficult to wear this module properly. Figure 6 shows that HD displacements are monotonically related to hand aperture. The main undesired effect was the tilting of the HD shell away from the hand dorsum, which (with reference to Figure 3-right) can be interpreted as a rotation of the *GHI* plane around the *y* axis (lifting marker *I* away from the hand dorsum); this was because of the moment reactions coming from the other modules during extension of the fingers, and resulted in a negative increment of the *z* coordinate and a positive increment of the *x* coordinate of the *J* marker.

In conclusion, we have show the statistically significant reliability of HX, through a quantitative analysis of hand and exoskeleton relative movements, especially concerning the finger modules. On the basis of the presented results, future development of HX will focus on refining the fixtures of the HD module, to stabilize its adherence. In the typical “active” operative mode, the displacements at the distal extremities were similar to those registered without motions, and fairly close to the averaged noise level, suggesting that the device can be effective in functional grasp actuation for weak hands. Future studies will also deal with the improvement of the sheath-cable transmission system, limiting friction losses in joint design.

## Authors’ original submitted files for images

Below are the links to the authors’ original submitted files for images. Authors’ original file for figure 1 Authors’ original file for figure 2 Authors’ original file for figure 3 Authors’ original file for figure 4 Authors’ original file for figure 5 Authors’ original file for figure 6

## Abbreviations

HX: hand exoskeleton device
DoFs: degrees of freedom
MCP: metacarpo-phalangeal joint
IP: inter-phalangeal joint
DIP: distal-inter-phalangeal joint
PG: palmar grasp
PN: pinch grip
KG: key grip
TH: thumb finger module of HX
IF: index finger module of HX
HD: hand dorsum module of HX
CI-95%: confidence interval at 95%
SD: standard deviation.
