# Supplementary figures and images for: Analysis of relative displacement between the HX wearable robotic exoskeleton and the user’s hand
Source: J Neuroeng Rehabil. 2014 Oct 18;11:147. doi: 10.1186/1743-0003-11-147 (PMC4271474; doi:10.1186/1743-0003-11-147)

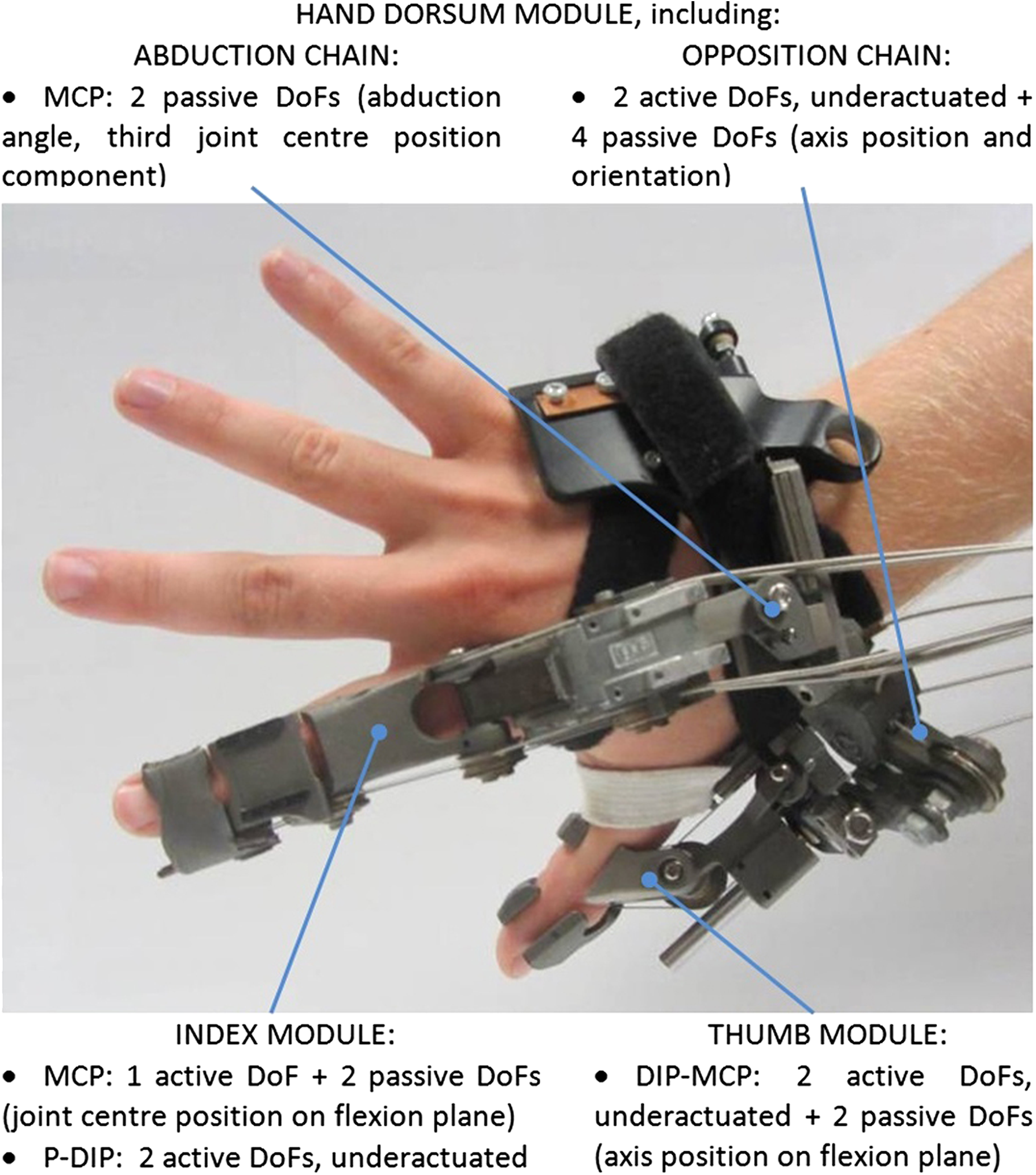

Supplement: Supplementary file 1 — Authors’ original file for figure 1 [file 12984_2014_678_MOESM1_ESM.tif]

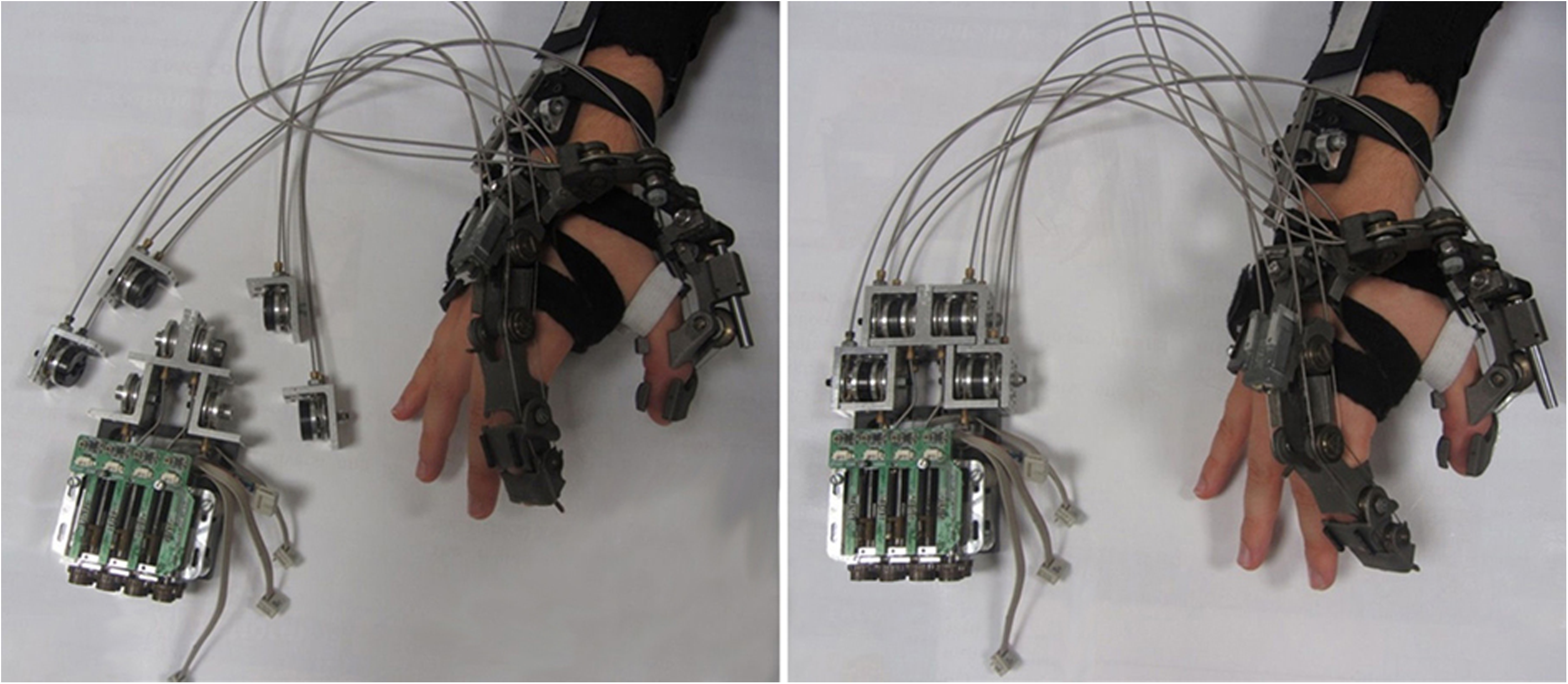

Supplement: Supplementary file 2 — Authors’ original file for figure 2 [file 12984_2014_678_MOESM2_ESM.tif]

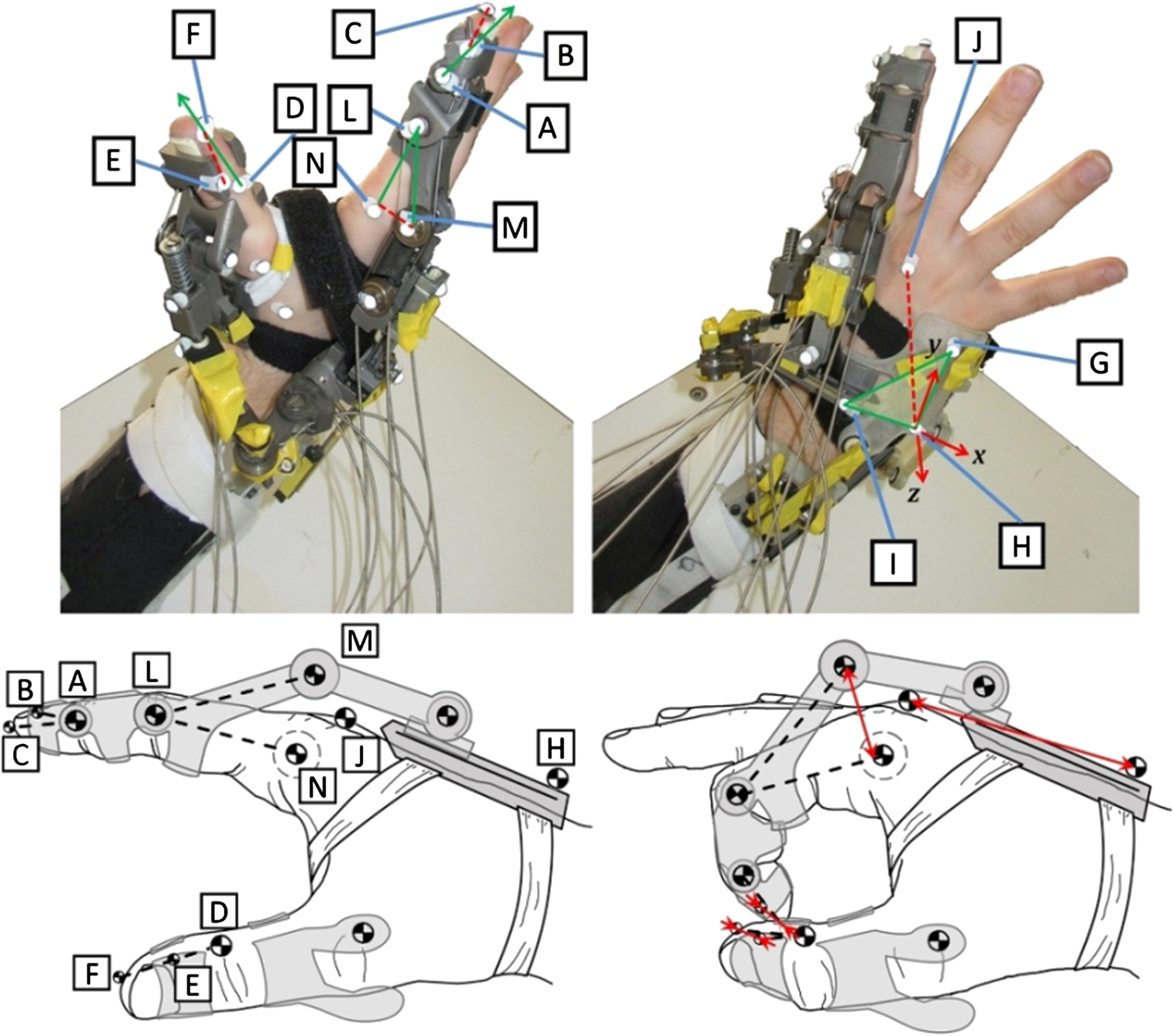

Supplement: Supplementary file 3 — Authors’ original file for figure 3 [file 12984_2014_678_MOESM3_ESM.tif]

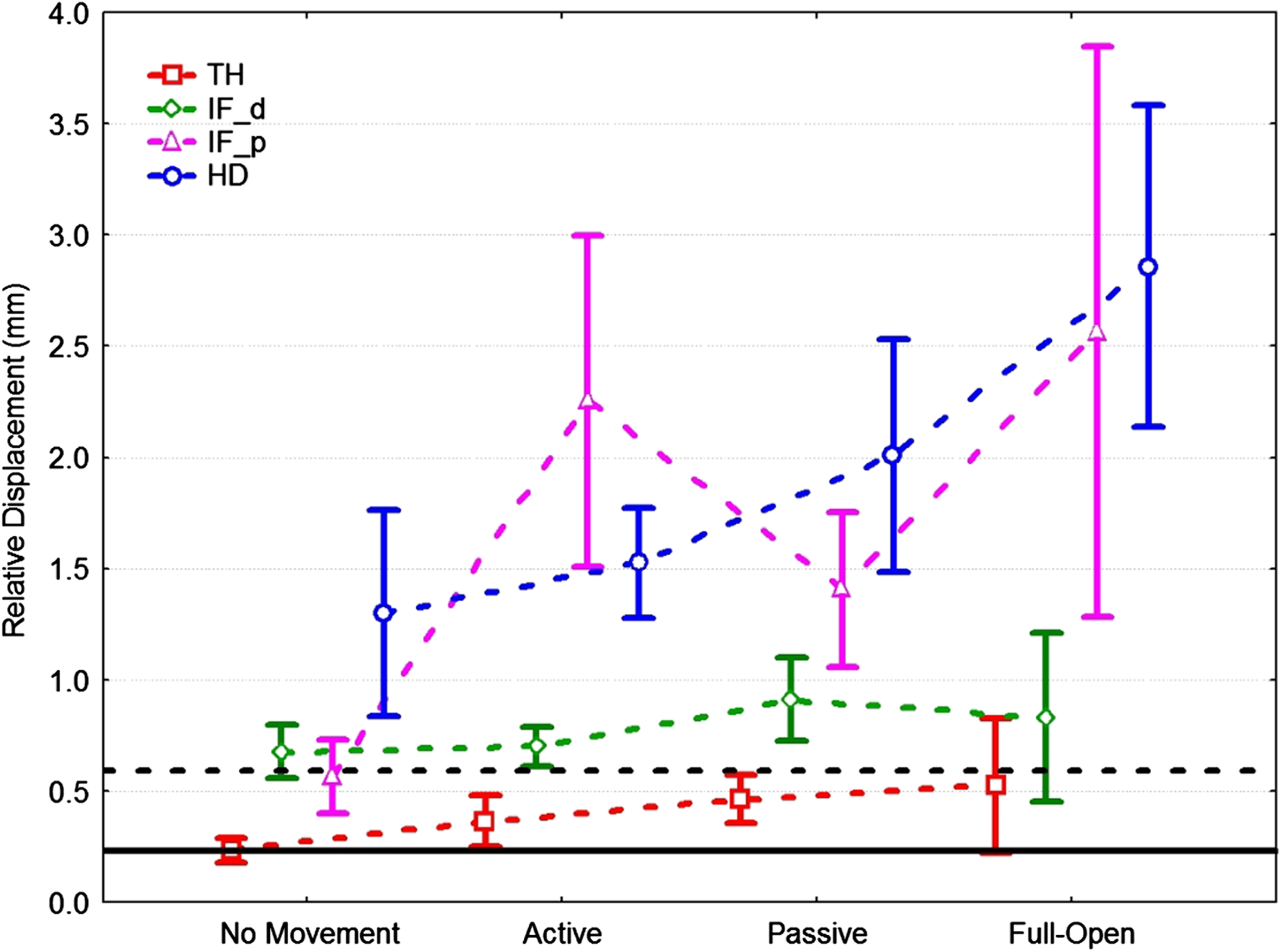

Supplement: Supplementary file 4 — Authors’ original file for figure 4 [file 12984_2014_678_MOESM4_ESM.tif]

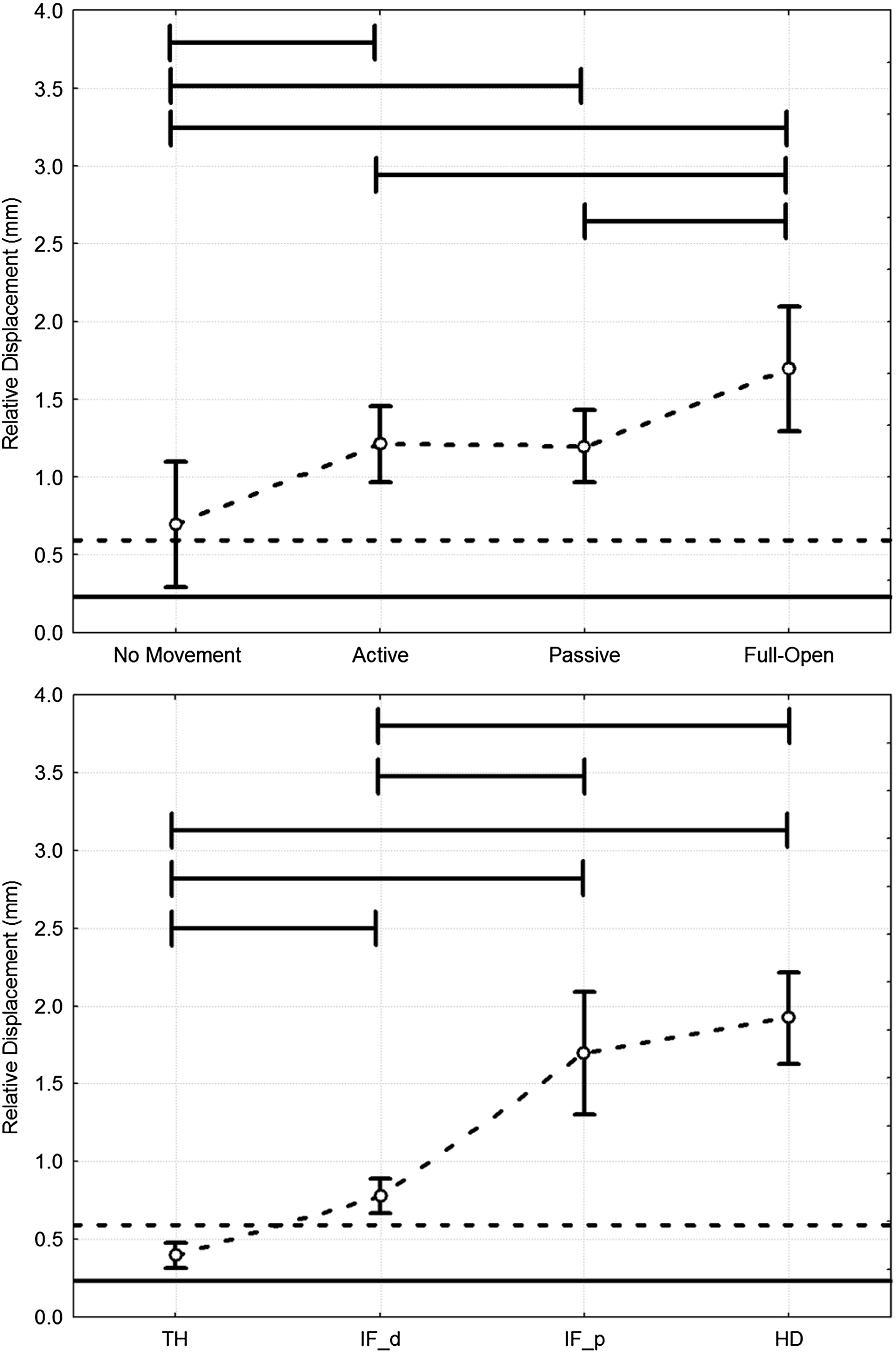

Supplement: Supplementary file 5 — Authors’ original file for figure 5 [file 12984_2014_678_MOESM5_ESM.tif]

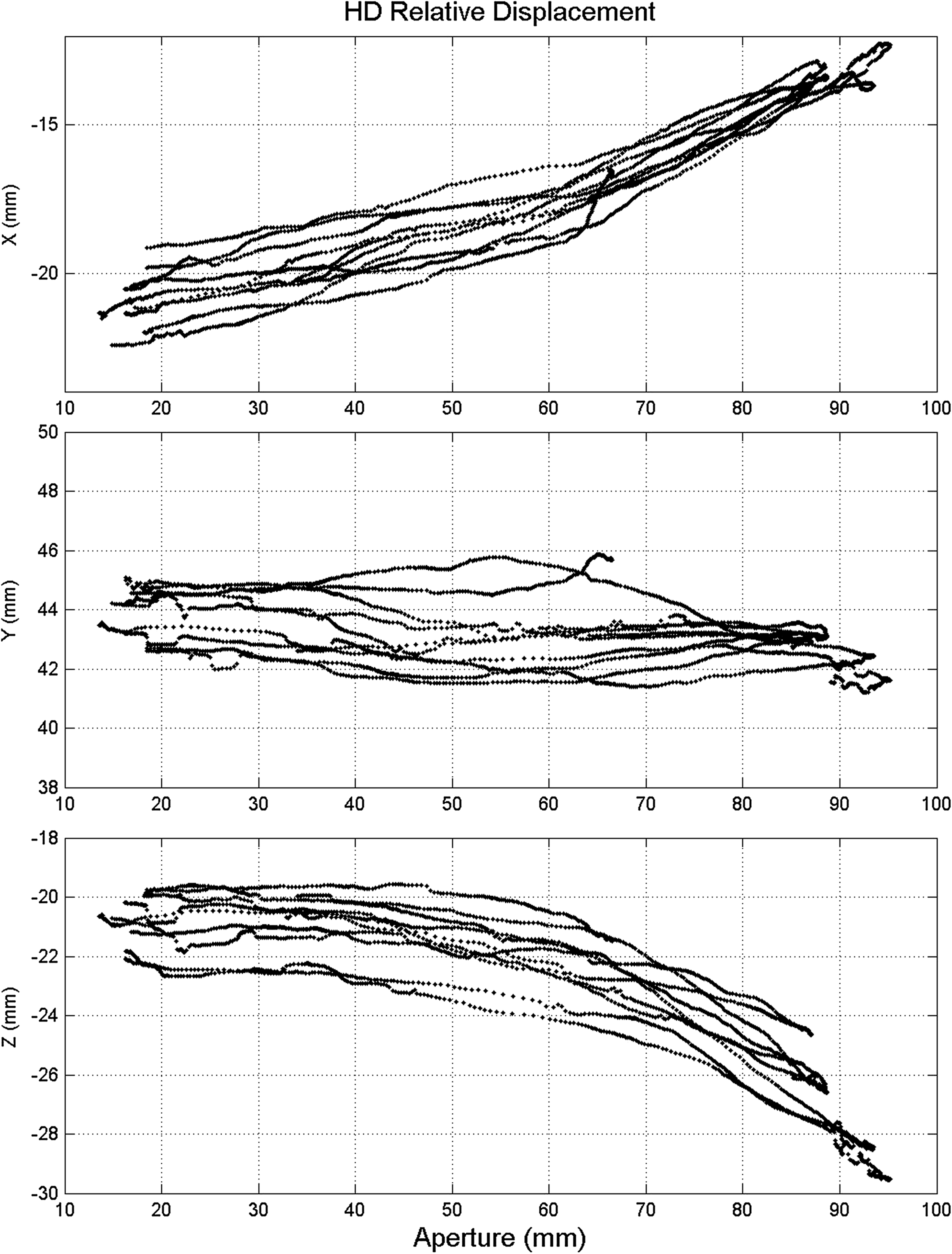

Supplement: Supplementary file 6 — Authors’ original file for figure 6 [file 12984_2014_678_MOESM6_ESM.tif]
